# A Rare Case of Progressive Palsy of the Lower Leg during Percutaneous Endoscopic Lumbar Discectomy via a Transforaminal Approach

**DOI:** 10.1155/2018/7803529

**Published:** 2018-01-31

**Authors:** Hiroaki Manabe, Kosaku Higashino, Kosuke Sugiura

**Affiliations:** Orthopedic Surgery, Takamatsu Municipal Hospital, 2-36-1 Miyawaki-cho, Takamatsu, Kagawa 760-8538, Japan

## Abstract

Percutaneous endoscopic discectomy (PED) for lumbar disc herniation is gaining popularity with the transforaminal (TF) approach preferred because it allows surgery under local anesthesia and preserves the spinal muscles. Although this procedure has some characteristic complications, it is rare for PED to be converted to conventional open surgery due to worsening of symptoms intraoperatively. Here, we report PED via the TF approach that required conversion to open surgery. A 20-year-old man with a large disc herniation at L3/4 developed severe progressive leg pain and muscle weakness of the left leg intraoperatively. Magnetic resonance imaging revealed that the size of the herniation was unchanged and the endoscope did not reach the herniated mass. We converted to open surgery, and the patient's postoperative course was favorable. We discuss the reasons for failure of the approach and suggest planning for an appropriate foraminoplasty to avoid the potential need for conversion to open surgery.

## 1. Introduction

Lumbar disc herniation is a tear in the outer annulus fibrosus of an intervertebral disc and is classified according to whether or not the nucleus pulposus bulges out beyond the outer annulus fibrosus. Lumbar disc herniation is usually caused by age-related degeneration of the annulus fibrosus, but it may also occur in response to trauma, a lifting injury, or sports activity. Percutaneous endoscopic lumbar discectomy (PED) via a transforaminal approach is commonly performed under local anesthesia to treat this disorder and requires an 8 mm skin incision [1, 2]. PED is a minimally invasive disc procedure that preserves the back muscles [3, 4], but there have been reports of serious complications, including retroperitoneal hematoma and intracranial hypertension [1, 3]. However, it is rare for PED to be converted to conventional open surgery due to worsening of symptoms intraoperatively. Here, we report the rare case of a patient who developed progressive palsy of the lower leg during PED via the transforaminal approach.

## 2. Case Report

**Figure 1:**
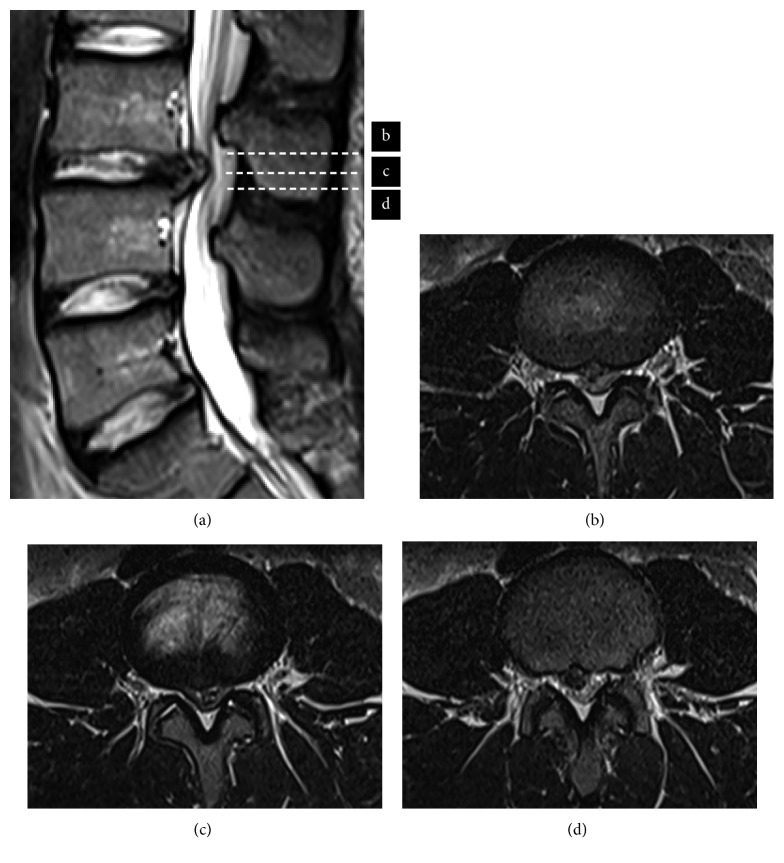
Preoperative sagittal T2-weighted MRI from sagittal view showed disc herniation at L3/4 disc level (a), and MRI from axial views showed left side huge herniation occupying the spinal canal (b–d).

A 20-year-old man presented to our hospital with a 6-month history of low back pain and a 2-month history of pain and numbness in both legs. He had intermittent claudication and could not tolerate being in the supine position. His symptoms had worsened despite conservative treatment. There was no muscle weakness. Hypoesthesia of the entire lower extremities was 3/10. On preoperative physical examination, the femoral nerve stretch test was positive, and the straight leg raise test was restricted to within 20° bilaterally. His preoperative visual analog scale (VAS) score was 1 cm for back pain and 8 cm for leg pain on both sides. Magnetic resonance imaging (MRI) and computed tomography (CT) after discography revealed a large disc herniation at L3/4 on the left side that was occupying 75% of the volume of the spinal canal (Figure 1).

## 3. Surgery

We planned for PED via the transforaminal approach under local anesthesia after obtaining informed consent. The surgical portal was created on the right side 8 cm from the longitudinal midline of the disc at L3/4, according to preoperative CT. The patient experienced no pain after the needle tip was advanced to the longitudinal midline of the disc at L3/4, but he suddenly developed severe pain in the right leg after insertion of the 8 mm cannula. We therefore changed the surgical portal to 7 cm from the longitudinal midline. However, an exiting L3 nerve root was confirmed under endoscopic view, and severe leg pain was evoked by stimulation using forceps. We then changed the surgical portal to 6 cm from the longitudinal midline, where it was just possible to position the cannula.

**Figure 2:**
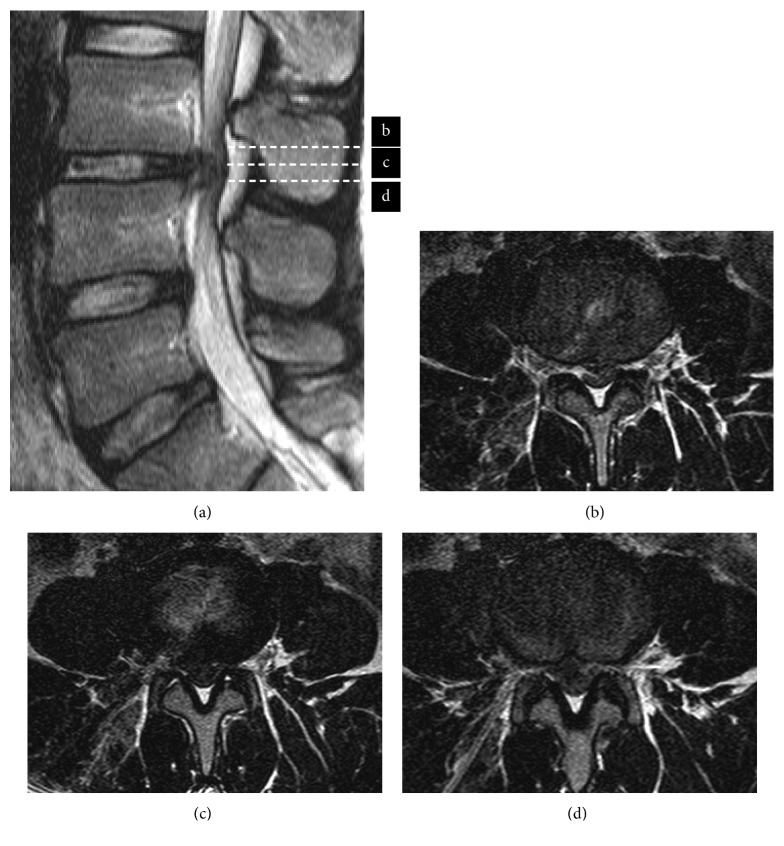
The first postoperative T2-weighted MRI did not change disc herniation at L3/4 disc level (a), and trajectory did not reach disc herniation (b–d).

After removal of the nucleus pulposus, the patient reported mild leg pain immediately upon introducing the scope via the inside-out method. Severe progressive leg pain developed when operation time exceeded 2 hours, and muscle weakness in the left lower limb became apparent. Manual muscle testing of the left quadriceps, extensor hallucis longus, tibialis anterior, flexor hallucis longus, and tibialis posterior was grade 3/5. The surgery was immediately stopped, and emergency MRI showed that the size of the herniation had not changed and that the endoscope did not reach the herniated mass (Figure 2). Informed consent for conversion to conventional open surgery was then obtained from the patient and his family. During the second surgery, the dura mater was found to be markedly compressed, and the L4 nerve root appeared tight under microscopic view. Both L4 nerve roots were released adequately after removal of the herniated mass.

## 4. Postoperative Course

**Figure 3:**
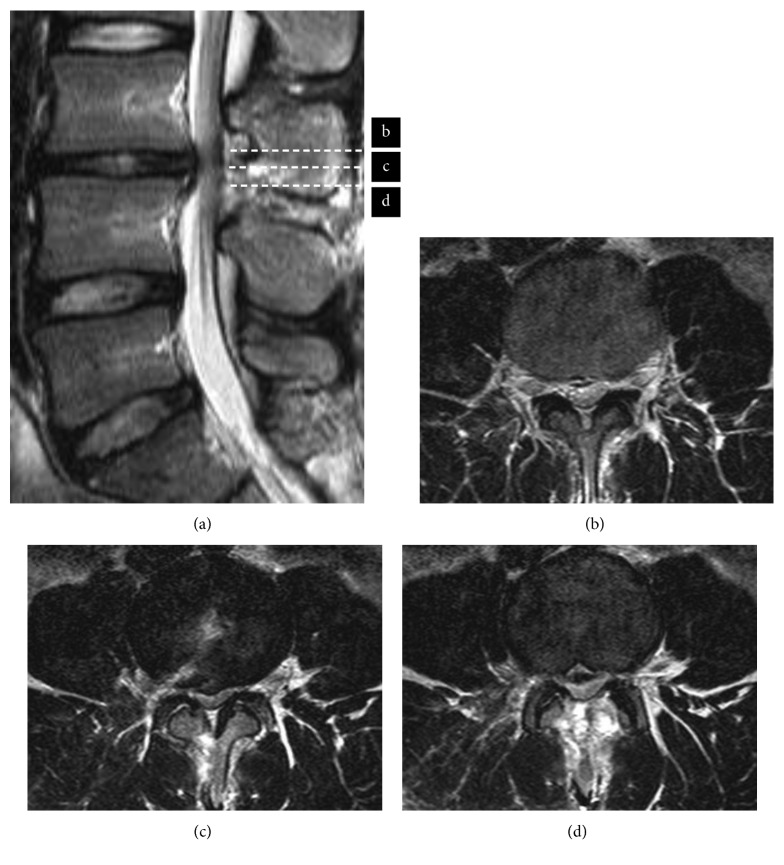
The latest postoperative T2-weighted MRI showed the removal of the nucleus pulposus and distension of the dura mater (a–d).

10 days after surgery, leg symptoms improved significantly after the open surgery, with a postoperative VAS score of 0 cm for back pain and 3 cm for each leg. Manual muscle tests results showed recovery to normal for all muscles, and the straight leg raise test was 60° without pain. MRI confirmed removal of the nucleus pulposus and showed distension of the dura mater (Figure 3).

## 5. Discussion

The advantages of PED via the transforaminal approach include avoiding injury to the back muscles and performing surgery under local anesthesia. However, a complication of this approach is exiting nerve root injury [5]. It is rare for neurological symptoms to worsen during PED, and any muscle weakness needs urgent spinal nerve decompression surgery. Some reports described that operative failure cases of PED showed the high grade canal compromise exceeding 50% [6, 7].

There are reports that foraminoplasty is effective for preventing exiting nerve root injury during PED via the transforaminal approach [8]. In our patient, foraminoplasty using a high-speed bur to enlarge the foramen might have avoided irritation of the exiting nerve root. After an appropriate foraminoplasty, it would have been possible to insert the cannula without stimulating or injuring the exiting nerve root [8]. In our case, we had not planned an appropriate foraminoplasty, so we needed to convert to conventional open surgery.

When a patient feels pain or numbness related to compression of an exiting nerve root during PED, it is essential that an appropriate foraminoplasty be performed using a high-speed bur to enlarge the foramen.

## 6. Conclusion

We have encountered this rare case of muscle weakness appearing during PED via the transforaminal approach. If a patient develops exiting nerve root symptoms in response to compression by the cannula during the procedure, a foraminoplasty may be effective and should be planned for.
